# Boosting Cosmeceutical Peptides: Coupling Imidazolium-Based Ionic Liquids to Pentapeptide-4 Originates New Leads with Antimicrobial and Collagenesis-Inducing Activities

**DOI:** 10.1128/spectrum.02291-21

**Published:** 2022-08-11

**Authors:** Ana Gomes, Lucinda J. Bessa, Iva Fernandes, Luísa Aguiar, Ricardo Ferraz, Cláudia Monteiro, M. Cristina L. Martins, Nuno Mateus, Paula Gameiro, Cátia Teixeira, Paula Gomes

**Affiliations:** a LAQV-REQUIMTE, Departamento de Química e Bioquímica, Faculdade de Ciências, Universidade do Porto, Porto, Portugal; b Centro de Investigação Interdisciplinar Egas Moniz (CiiEM), Egas Moniz - Cooperativa de Ensino Superior, Almada, Portugal; c Ciências Químicas e das Biomoléculas – CISA, Escola Superior de Saúde, Politécnico do Porto, Porto, Portugal; d i3S - Instituto de Investigação e Inovação em Saúde, Universidade do Porto, Porto, Portugal; e INEB - Instituto de Engenharia Biomédica, Porto, Portugal; f ICBAS, Instituto de Ciências Biomédicas Abel Salazar, Universidade do Porto, Porto, Portugal; Indian Institute of Science Bangalore

**Keywords:** antibacterial, antifungal, collagenesis-induction, cosmeceutical peptides, ionic liquid, Matrixyl, antimicrobial activity, collagen boosting, pentapeptide-4

## Abstract

Following our previous reports on dual-action antibacterial and collagenesis-inducing hybrid peptide constructs based on “pentapeptide-4” (PP4, with amino acid sequence KTTKS), whose *N*-palmitoyl derivative is the well-known cosmeceutical ingredient Matrixyl, herein we disclose novel ionic liquid/PP4 conjugates (IL-KTTKS). These conjugates present potent activity against either antibiotic-susceptible strains or multidrug resistant clinical isolates of both Gram-positive and Gram-negative bacterial species belonging to the so-called “ESKAPE” group of pathogens. Noteworthy, their antibacterial activity is preserved in simulated wound fluid, which anticipates an effective action in the setting of a real wound bed. Moreover, their collagenesis-inducing effects *in vitro* are comparable to or stronger than those of Matrixyl. Altogether, IL-KTTKS exert a triple antibacterial, antifungal, and collagenesis-inducing action *in vitro*. These findings provide solid grounds for us to advance IL-KTTKS conjugates as promising leads for future development of topical treatments for complicated skin and soft tissue infections (cSSTI). Further studies are envisaged to incorporate IL-conjugates into suitable nanoformulations, to reduce toxicity and/or improve resistance to proteolytic degradation.

**IMPORTANCE** As life expectancy increases, diseases causing chronic wound infections become more prevalent. Diabetes, peripheral vascular diseases, and bedridden patients are often associated with non-healing wounds that become infected, resulting in high morbidity and mortality. This is exacerbated by the fact that microbes are becoming increasingly resistant to antibiotics, so efforts must converge toward finding efficient therapeutic alternatives. Recently, our team identified a new type of constructs that combine (i) peptides used in cosmetics to promote collagen formation with (ii) imidazolium-based ionic liquids, which have antimicrobial and skin penetration properties. These constructs have potent wide-spectrum antimicrobial action, including against multidrug-resistant Gram-positive and Gram-negative bacteria, and fungi. Moreover, they can boost collagen formation. Hence, this is an unprecedented class of lead molecules toward development of a new topical medicine for chronically infected wounds.

## INTRODUCTION

The treatment of complicated skin and soft tissue infections (cSSTI) requires debridement or incision and drainage, complemented with antibiotic therapy. The guidelines for cSSTI treatment recommend the administration of systemic antibiotics that are effective against *methicillin-resistant* Staphylococcus aureus (MRSA) strains, which are among the multidrug-resistant (MDR) pathogens that prevail in health care facilities, followed by an antibiotic therapy program based on the culture assessments for a definitive treatment (1). However, resistance to the available antibiotics is rapidly increasing and currently widespread to many different species of Gram-positive and Gram-negative bacteria. Likewise, fungal infections are becoming more difficult to treat due to the development and spread of resistance among pathogenic fungi.

Peptide-based antimicrobials, like the cyclic lipopeptide daptomycin, offer clinicians an alternative to tackle MDR bacteria in hospital settings, but daptomycin is exclusively active against Gram-positive species, including MRSA (2). However, most cSSTI are of polymicrobial nature, also involving Gram-negative bacteria and fungi, meaning that new options are urgently needed to cope with the emerging post-antibiotic era (3). Fungal colonization of non-healing wounds has been traditionally neglected (4), but Kalan et al. have highlighted the importance of identifying not only bacterial, but also fungal species involved in cSSTI (4), namely, *Cladosporidium* spp. and *Candida* spp., including C. albicans and C. parapsilosis (5).

The healing process in cSSTI is often delayed or even impaired not only by the microbial infection itself, including biofilm formation in the wound bed, but also by other comorbidities such as diabetes, renal failure, vascular, neuropathic, and genetic disorders, or simply aging (6). In such cases, effective treatment of cSSTI must both quell infection and promote a fast and correct healing. In this regard, collagen, as a structural protein from the extracellular matrix (ECM), plays an important role in key steps of wound healing and closure (7). Because collagen is an endogenous protein, it is regarded as a desirable component for the development of biocompatible and biodegradable wound dressings/biomaterials (8). Along with collagen itself, collagen-derived/inspired peptides, such as cryptic collagen peptides (9), have been also considered as potential promoters of cell migration and proliferation, capable of inducing fibroblasts to produce new collagen, and consequently promoting faster wound healing (10). This motivated the development of the so-called matrikine peptides, and derivatives thereof, for topical application in the treatment of cSSTI, acting by promoting faster wound closure. Matrikine peptides are small peptide fragments that result from proteolysis of ECM macromolecules like collagen or elastin, with diverse potential biomedical applications, including in cosmetics (11, 12). For instance, “pentapeptide-4” (or PP4, with amino acid sequence KTTKS) is a widely studied matrikine that derives from type I human collagen, and is the smallest peptide sequence known to retain a potent ability to stimulate ECM (collagen and fibronectin) production (13, 14). The *N*-palmitoylated form of KTTKS, known as “palmitoyl pentapeptide-4” or Matrixyl, is used in the cosmetics industry due to its ability to cause a skin-rejuvenation/anti-wrinkle effect, probably associated to a collagenesis-inducing action (15, 16).

The potential of PP4 to promote wound healing in the context of cSSTI has recently attracted our attention. Thus, given that this matrikine peptide is devoid of antimicrobial action, we have developed a chimeric peptide where the KTTKS sequence was conjugated to an antimicrobial peptide (AMP), and which displayed potent (i) antibacterial activity against reference and MDR bacteria from clinical isolates; (ii) antibiofilm action; and (iii) a collagenesis-inducing effect comparable to that of Matrixyl (17). Further *N*-terminal modification of the aforementioned chimeric peptide with an imidazolium-based ionic liquid (IL) afforded an equally potent antimicrobial construct with increased stability toward enzyme-mediated modification (18). Indeed, IL are becoming quite attractive for biomedical applications, given their unique physicochemical characteristics, low cost, and high structural diversity that enables the synthesis of a wide panoply of different IL which can be easily tuned to meet specific requirements, including broad spectrum activity against bacteria (19) and fungi (20). Recently, alkylimidazolium-based IL have been proposed as an alternative antibacterial treatment for cSSTI focusing on Gram-positive pathogens (21). In addition, several IL or alkylimidazolium-derived IL have been found to improve the skin permeation of drugs (22–24), including ceftazidime, an antibiotic possessing poor water solubility and low skin permeation (25).

In view of the above, we have now investigated if the direct coupling of alkylimidazolium-based IL to the non-antimicrobial KTTKS sequence would afford a new type of peptide-based construct displaying collagenesis-inducing and antimicrobial action, despite not harboring an AMP motif. To this end, three different alkylimidazole-based IL were chemically modified to introduce the alkyne moiety required for subsequent coupling to different azide derivatives of KTTKS. Seven different IL-KTTKS conjugates were produced, and their antibacterial, antifungal, and collagenesis-inducing properties studied, as herein reported and discussed.

## RESULTS

### Synthesis of the target conjugates.

**FIG 1:**
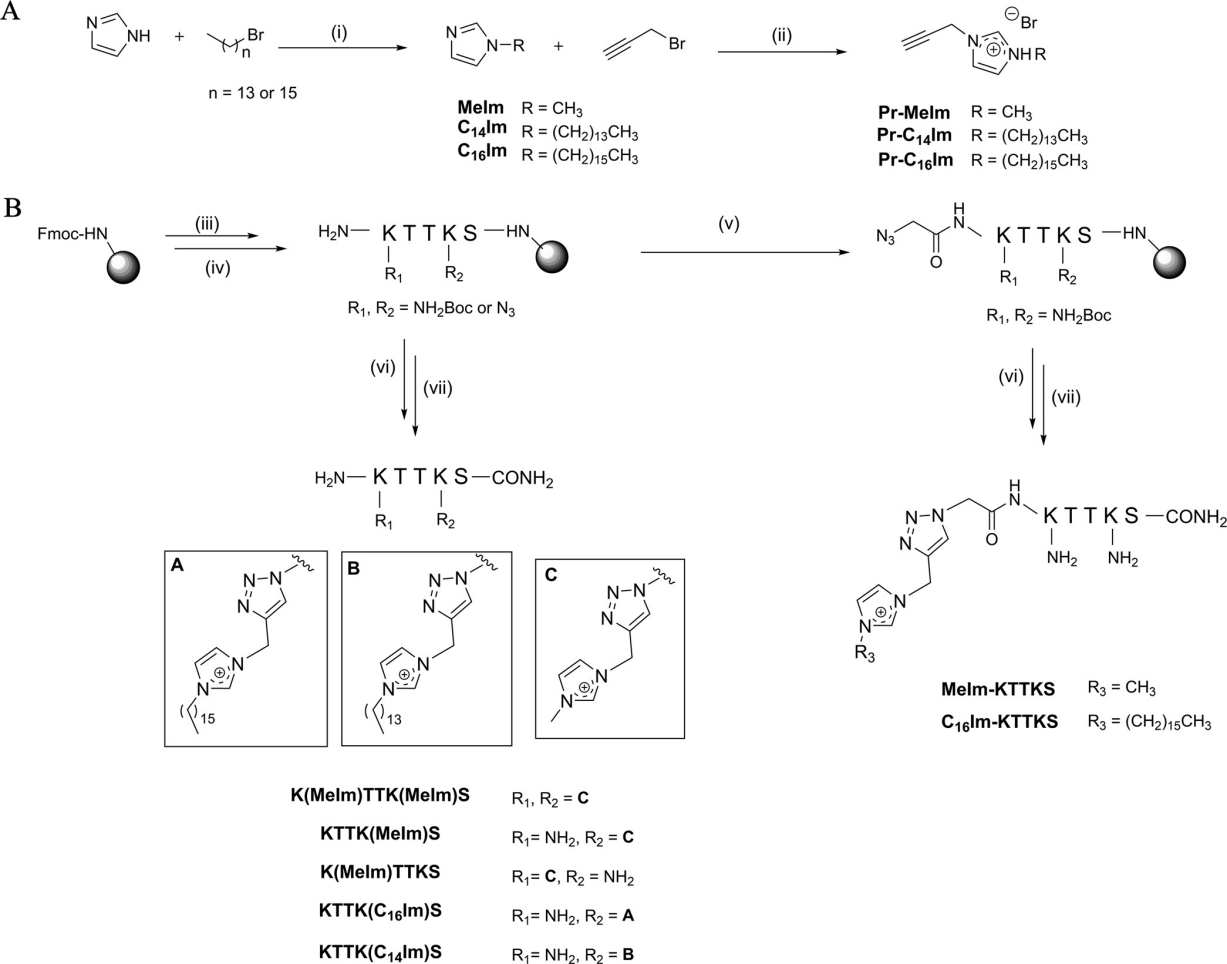
Route to the target IL-KTTKS conjugates. (A) Synthesis of the alkyne derivatives of the IL: (i) 1 molar equivalent (eq) of imidazole, 1.5 eq of potassium hydroxide, 1-bromotetradecane or 1-bromohexadecane in dimethyl sulfoxide (DMSO), 70°C, 5 h; (ii) 1.1 eq of C_16_Im, C_14_Im or MeIm and 1.0 eq of propargyl bromide (80% in toluene), 40°C, 24 h. (B) Synthesis of the azide derivatives of KTTKS and their coupling to the alkynyl-IL *via* CuAAC: (iii) 5 eq of Fmoc-protected amino acid, 10 eq of *N*-ethyl-*N*,*N*-diisopropylamine (DIEA) and 5 eq of *O*-(benzotriazol-1-yl)-*N*,*N*,*N*′,*N*′-tetramethyluronium hexafluorophosphate (HBTU) in *N*,*N*-dimethylformamide (DMF), 1 h, room temperature (r.t.); (iv) 20% piperidine in DMF, 15 min, r.t.; (v) 5 eq of azido acetic, 10 eq of DIEA, 5 eq of HBTU, 1 h, r.t.; (vi) 10 eq of DIEA, 10 eq of 2,6-lutidine, 1 eq of copper(I) bromide, 1 eq of sodium *L*-ascorbate and 1 eq of either Pr-MeIm, Pr-C_16_Im, or Pr-C_14_Im, in DMF:acetonitrile (ACN) (3:1 vol/vol), 24 h, r.t.; (vii) trifluoroacetic acid (TFA)/triisopropylsilane (TIS)/distilled water (95:25:2.5 vol/vol/vol).

The route toward the target IL-KTTKS conjugates started by the synthesis of the alkyne-modified imidazolium IL (Fig. 1A). Briefly, 1-bromohexadecane and 1-bromotetradecane were first reacted with imidazole, following a procedure previously described by Colonna et al. (step i, Fig. 1A) (26), to afford 1-tetradecyl-imidazole (C_14_Im) and 1-hexadecylimidazole (C_16_Im), respectively. After confirming the structures of both C_14_Im and C_16_Im by Nuclear Magnetic Resonance (^1^H- and ^13^C-NMR), these imidazoles were reacted with propargyl bromide according to Hu et al. (step ii, Fig. 1A) (27), to afford the three target imidazolium ILs, propargyl-MeIm (Pr-MeIm), propargyl-C_14_Im (Pr-C_14_Im) and propargyl-C_16_Im (Pr-C_16_Im). The structures of these ILs were confirmed by ^1^H-NMR, ^13^C-NMR, and electrospray ionization-ion trap mass spectrometry (ESI-IT MS).

**FIG 2:**
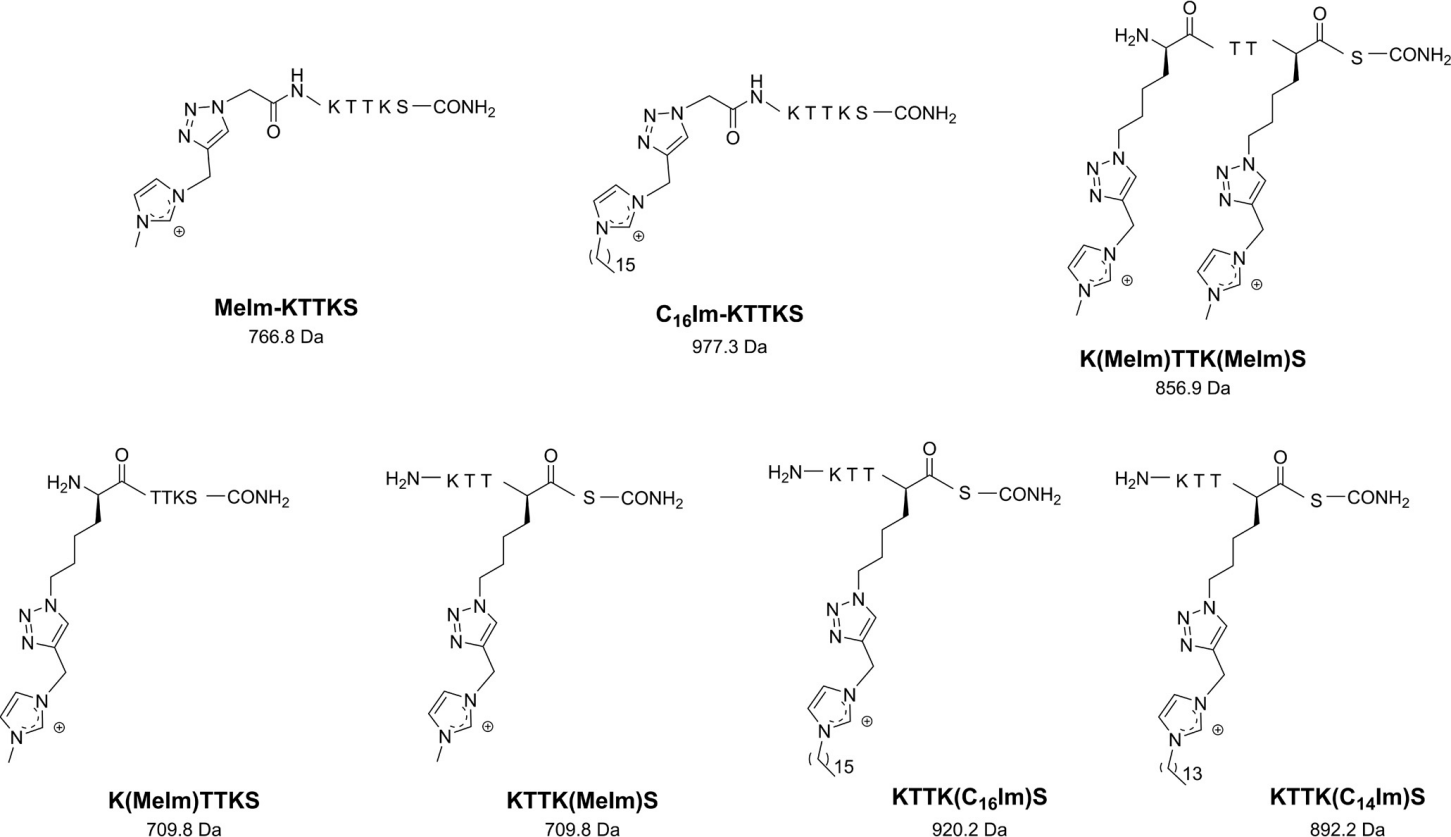
Structure of IL-KTTKS conjugates and corresponding molecular weight (in Da). The amino acids are represented in single letter code as defined by the IUPAC-IUBMB guidelines on nomenclature and symbolism for amino acids and peptides; exception is made to the amino acid residues whose side chain was coupled to ionic liquids *via* click chemistry, in which case the full modified structure is shown.

In parallel, conveniently modified derivatives of PP4 (amino acid sequence KTTKS) were produced by solid phase peptide synthesis (SPPS), to afford diverse final IL-KTTKS conjugates (Fig. 2) that differed in the: (i) propargyl-imidazolium building blocks used, (ii) insertion site of the latter (*N*-terminus, side chain of either or of both lysine residues), and (iii) length of the spacer between the imidazolium moiety and the peptide’s *N*-terminus. To this end, the PP4 sequence was first assembled, according to steps iii and iv in Fig. 1B, and conveniently protected lysine (Fmoc-Lys(Boc)-OH) or azido-lysine (Fmoc-Lys(N_3_)-OH) building blocks were inserted in the respective positions of the sequence, according to the desired site for the subsequent introduction of the imidazolium moiety *via* “click” copper(I)-catalyzed alkyne-azide cycloaddition (CuAAC). To produce the peptides modified at the *N*-terminus, the sequence bearing two natural Lys residues was assembled and further elongated through coupling of azido acetic acid (step v, Fig. 1B) yielding a 2- carbon (ethyl) spacer between the *N*-terminal lysine and the imidazolium moiety to be incorporated *via* CuAAC. This click reaction was next performed on-resin on all precursor azido-peptides, using the desired propargyl-imidazolium IL (step vi, Fig. 1B) and CuAAC conditions previously reported by us (18). After acidolytic cleavage (step vii, Fig. 1B) and purification of the crude conjugates thus obtained by reverse-phase preparative high performance liquid chromatography (RP-HPLC), all the resulting IL-KTTKS conjugates were isolated in high purity (>95%), and their expected molecular weights confirmed by ESI-IT MS.

In addition to the target conjugates, the reference cosmeceutical peptide Matrixyl (C_16_-KTTKS*-OH*), its *C*-terminal carboxamide analogue (C_16_-KTTKS*-NH_2_*), and the native PP4 (KTTKS) were also assembled by SPPS, following procedures recently reported by us (17). For the palmitoylated peptides, after the full amino acid sequence of PP4 was assembled, palmitic acid (C_16_) was coupled. Then, acidolytic cleavage from the solid support delivered the crude peptides that were purified by RP-HPLC. The final peptides were obtained in high purity and their molecular weights confirmed by ESI-IT MS.

### Antibacterial activity *in vitro*.

The antimicrobial activity of the IL-KTTKS conjugates was assessed *in vitro* against reference bacterial strains (American Type Culture Collection, ATCC). The minimal inhibitory concentration (MIC) was determined according to the Clinical and Laboratory Standards Institute (CLSI) guidelines (28) against Gram-positive (S. aureus, Enterococcus faecalis) and Gram-negative (Escherichia coli, Pseudomonas aeruginosa) bacteria. The MIC values obtained are shown in Table 1. Notably, the reference peptides C_16_-KTTKS-*NH_2_* and C_16_-KTTKS-*OH* were respectively soluble in water and dimethyl sulfoxide (DMSO), but both precipitated when diluted in cation-adjusted Mueller-Hinton broth (MHB2), the culture medium recommended by the CLSI guidelines, which hampered the determination of the MIC values for these reference peptides. Data in Table 1 show, as expected, that the peptide KTTKS alone is devoid of significant antibacterial activity, and that MIC values for the reference 1-hexadecyl-3-methylimidazolium bromide ([C_16_ M1Im][Br]) IL are in agreement with those previously reported (29). Interestingly, all conjugates bearing methyl imidazolium (MeIm) units were inactive against the tested bacterial species, even at the highest concentrations used, regardless the number or position of the MeIm moieties in the overall structure. In turn, replacing the methyl substituent in the imidazolium ring by either a tetradecyl (C_14_) or a hexadecyl (C_16_) group, led to an improvement in the antibacterial activity, delivering MIC values from 6.45 to 52.6 μg/mL, hence adding antimicrobial activity to the parent KTTKS peptide. Given that KTTK(C_16_Im)S and C_16_Im-KTTKS showed the strongest antibacterial activities, and reflect two different conjugation positions, both these peptides were further investigated by determining their MIC against Staphylococcus epidermidis, Streptococcus pyogenes (both Gram-positive), and Klebsiella pneumoniae (Gram-negative), chosen due to their abundance in the skin (S. epidermidis) (30), relevance to cSSTI (S. pyogenes) (31–33), and relation to the so-called “ESKAPE” pathogens (K. pneumoniae) (34). The noncovalent mixture of the parent peptide KKTKS and the [C_16_ M1Im][Br] ionic liquid, presented MIC values comparable to those of [C_16_ M1Im][Br] alone.

**TABLE 1:** MIC values (*n* = 3) in μM (in μg/mL) of the IL-KTTKS conjugates against Gram-negative and Gram-positive bacteria (ATCC reference strains)

Peptide	MIC in μM (in μg/mL)						
	E. coli *AT*CC 25922	P. aeruginosa ATCC 27853	S. aureus *ATCC* 29213	E. faecalis ATCC 29212	*K. pneumonia A*TCC 138830	S. epidermidis ATCC 14990	S. pyogenes ATCC 19615
K(MeIm)TTKS	> 1030.1 (731.2)				ND^*a*^	ND^*a*^	ND^*a*^
KTTK(MeIm)S	> 954.2 (677.3)				ND^*a*^	ND^*a*^	ND^*a*^
K(MeIm)TTK(MeIm)S	> 1245.5 (1067.4)				ND^*a*^	ND^*a*^	ND^*a*^
KTTK(C_14_Im)S	29.5 (26.3)	58.9 (52.6)^*b*^	29.5 (26.3)	58.9 (52.6)^*b*^	ND^*a*^	ND^*a*^	ND^*a*^
KTTK(C_16_Im)S	7.0 (6.45)	32.5 (29.9)	14.0 (12.9)	32.5 (29.9)	53.8 (49.5)	5.4 (5.0)	10.9 (10.0)
MeIm-KTTKS	> 825.9 (633.4)				ND^*a*^	ND^*a*^	ND^*a*^
C_16_Im-KTTKS	14.3 (14.0)	28.7 (28.0)	14.3 (14.0)	28.7 (28.0)	27.4 (26.8)	9.5 (9.3)	18.9 (18.5)
KTTKS	>1820^*e*^				ND^*a*^	ND^*a*^	ND^*a*^
[C_16_ M1Im][Br]	60	>240	0.94^*d*^	0.94^*c*^	60	ND^*a*^	ND^*a*^
KTTKS:[C_16_ M1Im][Br] (1:1)	60	>240	0.94^*d*^	0.18^*c*^	60	ND^*a*^	ND^*a*^
Ciprofloxacin	0.012 (0.004)	0.18 (0.06)	1.5 (0.5)	0.38 (0.125)	0.75 (0.25)	0.75 (0.25)	6.04 (2.0)

a Not determined.

b The minimal bactericidal concentration (MBC) was 2× the MIC.

c MBC = 15 μM.

d MBC = 30 μM; in all other cases, the MBC was equal to the MIC.

e Value from (17).

The antibacterial activities of the best couple of conjugates, i.e., C_16_Im-KTTKS and KTTK(C_16_Im)S, and of the reference antibiotic ciprofloxacin, were further assessed against MDR clinical isolates of K. pneumoniae (KP010), S. aureus (SA007), and P. aeruginosa (PA004). MIC values thus obtained are displayed in Table 2 and show that both conjugates preserve their antibacterial activity observed against susceptible ATCC bacterial strains. Relevantly, the conjugates were clearly more active than the reference antibiotic ciprofloxacin against the MDR isolates; for instance, the MIC value obtained for C_16_Im-KTTKS against SA007 is nearly 10-fold higher than that of ciprofloxacin.

**TABLE 2:** MIC values (*n* = 3) in μM (in μg/mL) for C_16_-Im-KTTKS and KTTK(C_16_Im)S against MDR clinical isolates of Gram-positive and Gram-negative bacteria

MDR	Peptide		Ciprofloxacin
	C_16_Im-KTTKS	KTTK(C_16_Im)S	
KP010	37.9 (37.0)	21.7 (20.0)	48.0 (16.0)
PA004	18.9 (18.5)		96.0 (32.0)
SA007	18.9 (18.5)^*a*^		193.0 (64.0)

a The MBC was 2× the MIC; In all other cases the MBC was equal to the MIC.

The antibacterial activity of C_16_Im-KTTKS and KTTK(C_16_Im)S was also assessed in simulated wound fluid (SWF) (35) against S. aureus (ATCC 29213), to check if it was preserved in this medium. MIC values were obtained in both SWF and MHB media in three independent experiments run in triplicates (Table 3), and indicated that the antibacterial activity in SWF was the same as that in MHB for C_16_Im-KTTKS, and decreased for KTTK(C_16_Im)S displaying a MIC twice as high.

**TABLE 3:** MIC (MBC) values (*n* = 3) in μg/mL for C_16_Im-KTTKS and KTTK(C_16_Im)S against S. aureus (ATCC 29213) in MHB and SWF

Peptide	MIC in μg/mL [MBC]	
	MHB	SWF
C_16_Im-KTTKS	16-32 [32 to 64]	16-32 [32 to >64]
KTTK(C_16_Im)S	32 [64 to 128]	64-128 [128 to >128]

### Antifungal activity *in vitro.*

The antifungal activity of the best couple of IL-KTTKS conjugates, their parent building blocks, and respective noncovalent 1:1 mixture, were all assessed against three species of *Candida*, namely, Candida albicans (ATCC 90028), Candida glabrata (ATCC 90030), and Candida parapsilosis (ATCC 22019). The MIC values were determined according to the European Committee on Antimicrobial Susceptibility Testing (EUCAST) protocol (36) and are shown on Table 4. Both conjugates, KTTK(C_16_Im)S and C_16_Im-KTTKS, were equally active against all *Candida* spp, with MIC values ranging from 2.4 to 5.4 μM. Both peptides were twice more active against C. parapsilosis than against the other two *Candida* species. Relevantly, the noncovalent mixture KTTKS:[C_16_ M1Im][Br] (1:1) showed a potent activity against all *Candida* spp, being equipotent to the parent IL alone, and both seven times more active than the reference antifungal drug fluconazole.

**TABLE 4:** MIC values (*n* = 2) in μM (in μg/mL) for the best performing conjugates, their parent building blocks, and respective 1:1 noncovalent mixture on ATCC *Candida* spp.

Peptide	MIC in μM (in μg/mL)		
	C. albicans ATCC 90028	C. glabrata ATCC 90030	C. parapsilosis ATCC 22019
KTTK(C_16_m)S	5.4 (5.0)	5.4 (5.0)	2.7 (2.5)
C_16_Im-KTTKS	4.7 (4.6)	4.7 (4.6)	2.4 (2.3)
KTTKS	>60	>60	>60
[C_16_M1Im][Br]	0.93	0.93	0.93
KTTKS:[C_16_ M1Im][Br] (1:1)	0.93	0.93	0.93
Fluconazole	1.6 (0.5)	26 (8)	6.5 (2)

### Toxicity to HFF-1 and HaCaT cells.

The cytotoxicity of KTTK(C_16_Im)S and C_16_Im-KTTKS conjugates was assessed on human foreskin fibroblasts (HFF-1) and human immortalized keratinocytes (HaCaT). The results shown in Table 5, are expressed as the conjugate concentration causing a 50% cell growth inhibition (IC_50_). As expected from previous reports (37), both the parent peptide sequence KTTKS and the derived reference cosmeceutical Matrixyl (C_16_-KTTKS-*OH*) did not show relevant toxicity against the cell lines tested, at up to 100 μM. In turn, the parent IL [C_16_ M1Im][Br] and its covalent equimolar mixture with PP4, KTTKS:[C_16_ M1Im][Br] (1:1), were significantly toxic. Interestingly, covalent conjugation of the peptide to the IL resulted in an intermediate situation, as conjugates were more toxic than the peptide alone, but clearly less toxic than the IL alone or than its noncovalent mixture with the peptide.

**TABLE 5:** IC_50_ values, in μM, obtained for the best performing conjugates, their parent building blocks, and respective noncovalent 1:1 mixture, against HFF-1 and HaCaT cells (after 24 h of incubation)

Peptide	IC_50_ ± SEM (μM)^*a*^	
	HaCaT	HFF-1
KTTK(C_16_Im)S	23.05 ± 1.10	12.71 ± 1.06
C_16_Im-KTTKS	36.60 ± 1.09	22.46 ± 1.08
C_16_-KTTKS-*OH*	>100	79.74 ± 2.82
KTTKS	>100	ND
[C_16_ M1Im][Br]	6.27 ± 1.02	ND
KTTKS:[C_16_ M1Im][Br] (1:1)	6.46 ± 1.01	8.47 ± 1.03

a Data expressed as mean ± SEM of 2 independent experiments (*n* = 8).

### Collagen production *in vitro.*

**FIG 3:**
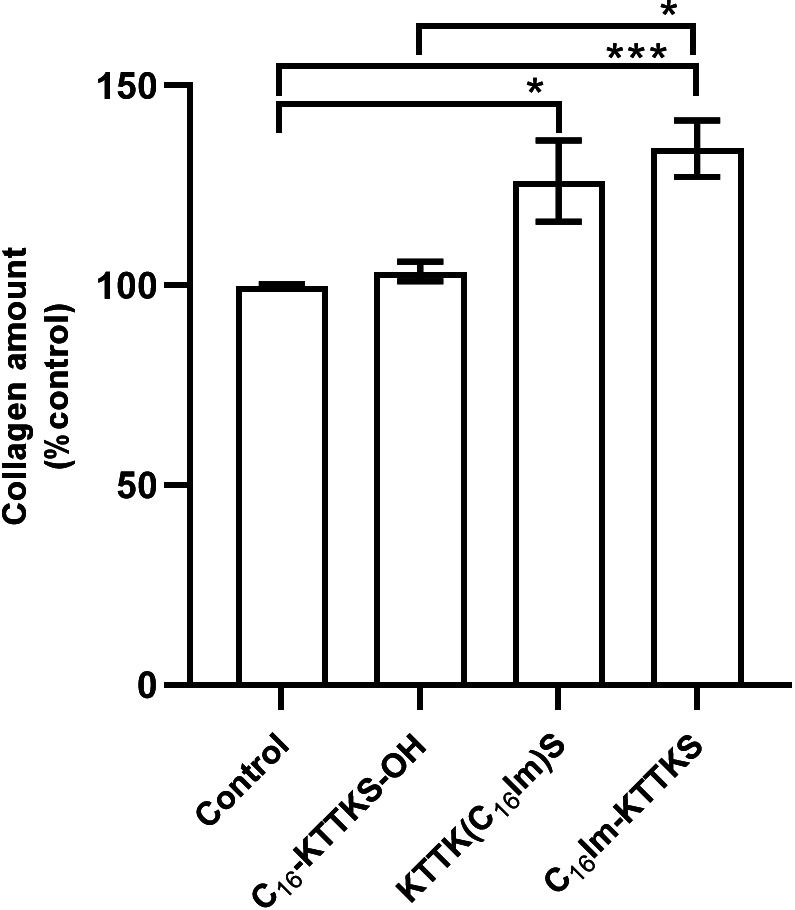
Collagen synthesis by HDF, in the presence of C_16_-KTTKS-*OH* (Matrixyl), KTTK(C_16_Im)S and C_16_Im-KTTKS at 5 μM, using the Sircol Kit. Data are presented as mean ± SEM (three independent experiments in triplicates) expressed in collagen amount (% of control); *, *P* < 0.05; **, *P* < 0.01.

The point of conjugating antimicrobial ILs to a collagenesis-inducing peptide was to afford a simple construct able to exert a dual antimicrobial and skin rebuilding action. Therefore, the two best IL-KTTKS conjugates were further tested for their ability to promote collagen production by human dermal fibroblasts (HDF) *in vitro*. This was assessed using the Sircol kit assay, whereby the amount of newly formed collagen in the ECM that is deposited in the microwell-plated cell cultures is solubilized in an acidic medium and next quantified through a collagen standard curve according to the Sircol kit assay procedure (38). Assays were conducted in different conditions for comparison, namely, in the presence of the reference cosmeceutical Matrixyl (positive control – C_16_-KTTKS-*OH*), of the test conjugates KTTK(C_16_Im)S and C_16_Im-KTTKS, and in the absence of any peptide (negative control). Data presented in Fig. 3 show that both conjugates induce HDF cells to produce more collagen, compared to the negative control. No significant difference was observed between both conjugates or between KTTK(C_16_Im)S conjugate and reference Matrixyl, demonstrating that the ability of Matrixyl to induce collagenesis is not affected by the introduction of the imidazolium IL at the Lys side chain of the peptide sequence.

## DISCUSSION

Recently, we have demonstrated that it is possible to produce a dual-action antimicrobial and collagenesis-inducing chimeric peptide, by combining the amino acid sequence of an AMP to that of the non-antimicrobial well-known cosmeceutical peptide PP4 (17). We were further able to show that such potent antimicrobial activity was preserved when coupling an imidazolium IL to the *N*-terminus of the chimeric peptide *via* the CuAAC “click” approach, which conferred the peptide resistance to enzyme-mediated modification (18). Based on these findings, and on the widely reported antimicrobial properties of imidazolium (and other) ILs (21, 39, 40), we hypothesized that it might be possible to preserve the dual antimicrobial and collagenesis-inducing activity by leaving the AMP sequence out and coupling the IL directly to the amino acid sequence of the cosmeceutical peptide PP4. The antibacterial activity of the new constructs herein presented, IL-KKTKS, was assessed against reference bacterial strains and results obtained allow us to advance a couple of structure-activity relationships (SAR) on the (i) IL insertion site, as the covalent graft was at either the *N*-terminus or the Lys1/Lys4 side chains of the KTTKS sequence, and (ii) length of the alkyl substituent in the imidazole ring, which was varied between one (methyl or Me spacer), 14 (tetradecyl or C_14_ spacer), and 16 (hexadecyl or C_16_ spacer) carbons. Hence, antibacterial activity (i) increases with the length of the alkyl substituents in the IL moiety [KTTK(C_14_Im)S *versus* KTTK(C_16_Im)S] and (ii) is depleted in all conjugates bearing the methyl-substituted imidazolium IL, regardless of other structural features. This alkyl chain length effect agrees with previous studies on antimicrobial ILs (29, 41). Moreover, MIC values were also determined for the parent building blocks KTTKS and [C_16_ M1Im][Br], as well as for their noncovalent equimolar mixture, indicated as KTTKS:[C_16_ M1Im][Br] (1:1), so that the importance of covalent conjugation could be assessed. The noncovalent mixture KTTKS:[C_16_ M1Im][Br] (1:1) presented MIC values similar to those of [C_16_ M1Im][Br] alone, confirming that the IL building block is mainly responsible for the activity observed for the mixture, as expected. Interestingly, when comparing the MIC values of the noncovalent mixture KTTKS:[C_16_ M1Im][Br] (1:1) with those of the covalent conjugates KTTK(C_16_Im)S and C_16_Im-KTTKS, it is apparent that covalent conjugation is clearly beneficial for activity against Gram-negative bacteria, but not so much against Gram-positive bacteria. Still, whereas the noncovalent mixture is bacteriostatic for Gram-positive species at MIC values, the covalent conjugates are bactericidal at these concentrations. These results indicate that the antibacterial activity of the covalent conjugates KTTK(C_16_Im)S and C_16_Im-KTTKS is not only modulated by the IL building block, but also by its conjugation to the peptide, eventually due to the CuAAC-mediated insertion of the 1,2,3-triazole ring that has been proven as an important antimicrobial pharmacophore (42). Hence, biophysical studies should be performed to further explore the mechanism(s) of action of IL-KTTKS conjugates. The conjugates KTTK(C_16_Im)S and C_16_Im-KTTKS also showed a potent activity against MDR clinical isolates of Gram-positive and Gram-negative bacteria, being more active than the reference antibiotic ciprofloxacin. This is a relevant finding, considering that the three clinical isolates tested refer to bacterial species belonging to the “ESKAPE” group of pathogens which encompasses life-threatening nosocomial pathogens, namely, **E**nterococcus faecium, **S**taphylococcus aureus, **K**lebsiella pneumoniae, **A**cinetobacter baumannii, **P**seudomonas aeruginosa, and **E**nterobacter spp. The ability of “ESKAPE” pathogens to escape the action of currently available antibiotics is one of the major health care threats of our times, especially for Gram-negative bacteria, against which the world is running out of effective options (43). Additionally, the conjugates showed to retain (C_16_Im-KTTKS) or slightly decrease (KTTK(C_16_Im)S) the antibacterial activity against S. aureus in SWF. The real wound fluids contain proteins, including growth factors and proteases, among other constituents, with a specific composition that depends on if it is a healing or a non-healing wound. In fact, the fluid formed in the open wound generally has wound-healing properties and is beneficial for a fast wound recovery, however in the presence of a high amount of certain components or of microorganisms, the healing process can be delayed or impaired (44). Moreover, the presence of metalloproteinases and other enzymes may rapidly inactivate any peptide-based wound treatments. As such, these preliminary observations using SWF, which mimics the wound exudate, are relevant as they are indicative that the IL-peptide conjugates, especially C_16_Im-KTTKS, can be more stable in the wound environment compared with analogues where the peptide is not protected at the *N*-terminus, which is in line with our previous observations regarding the stabilization effect of linking an ionic liquid to the *N*-terminus of a bioactive peptide (18).

The IL-KTTKS conjugates herein advanced have also shown interesting antifungal properties, especially against C. parapsilosis. This is one of the most common non-C. albicans species of *Candida*, which are regarded as important nosocomial pathogens of concern as they were reported to be involved in cases of sepsis (45) and cSSTI (5). In this regard, the antifungal activity of the conjugates was assessed, and MIC values observed were as low as 2.4 and 2.7 μM against C. parapsilosis and 4.7 and 5.7 μM against C. albicans. This concurs with recent findings by Reddy et al., showing that imidazolium ILs with long alkyl chains, e.g., dodecyl and hexadecyl, have potent activity against C. albicans (20). Still, as observed in antibacterial activity assays, the non-covalent mixture and the parent IL are more potent than the IL-KTTKS conjugates against *Candida* spp., which indicates that the antifungal activity of the covalent conjugates is not only modulated by the IL building block, but also by their conjugation to the peptide (devoid of antifungal activity). Therefore, further biophysical studies should be performed to shed some light into possible mechanism(s) of action of IL-KTTKS conjugates against *Candida* species.

The evaluation of the cytotoxicity of the IL-KTTKS conjugates is obviously important on its own to check for selectivity, but also due to the toxicity effects often associated to IL, depending on, e.g., cation alkyl chain length (46) or specific ions used (47). The parent IL [C_16_ M1Im][Br] and its covalent equimolar mixture with the peptide, KTTKS:[C_16_ M1Im][Br] (1:1), were significantly toxic against the human cell lines tested, in agreement with previous reports on ILs (19). Therefore, covalent conjugation of the IL to the peptide confers on the one hand, antimicrobial activity to an otherwise peptide building block devoid of such activity and, on the other, reduced cytotoxicity compared to the parent IL.

The conjugates C_16_Im-KTTKS and KTTK(C_16_Im)S were further assessed for their ability to induce collagen production by HDF *in vitro*. The conjugates showed to be comparable to the reference cosmeceutical Matrixyl and more active than the control. The mechanism behind the collagen inducing effect of Matrixyl is not well explored, although Jones et al. have suggested that the self-assembling properties of the Matrixyl amphiphilic structure favors recognition of pro-collagen residues, *via* formation of nanotapes that expose relevant peptide epitopes (48). Irrespective of that, no significant difference between both conjugates were observed, indicating that changing the side chain of Lys4 did not affect the peptide’s collagenesis-inducing behavior.

Altogether, our findings are unprecedented as well as remarkable, as they provide confirmation or our initial hypothesis, by advancing a couple of IL-peptide conjugates, C_16_Im-KTTKS and KTTK(C_16_Im)S, that possess antibacterial, antifungal, and collagenesis-inducing activity *in vitro*, the latter being actually comparable with that of the cosmeceutical ingredient Matrixyl based on the KTTKS peptide. Further, the site of insertion of the IL does not significantly affect the overall *in vitro* properties of the conjugates [C_16_Im-KTTKS *versus* KTTK(C_16_Im)S], although *N*-terminal conjugation seems to better preserve the conjugates’ antibacterial action in SWF and to improve the collagen synthesis by HDF. However, further studies are envisaged to incorporate IL-conjugates into nanoformulations, which will reduce toxicity and/or improve resistance to proteolytic degradation. Moreover, because the IL-KKTKS conjugates will be applied in the treatment of cSSTI, which are mainly polymicrobial infections, the antimicrobial activity of IL-KTTKS on polymicrobial cultures will be further investigated. This will enable selection of best IL-KTTKS based formulations to advance for *in vivo* studies.

Considering that the KTTKS and other small collagenesis-boosting peptides are already produced at industrial scale as ingredients for cosmetic products, this report unveils the value of IL-cosmeceutical peptide conjugates as a promising start for future development of cost-effective topical formulations for the treatment of skin disorders, from mild to severe ones like cSSTI.

## MATERIALS AND METHODS

### Chemical synthesis.

**(i) 1-tetradecylimidazole (C_14_-Im) and 1-hexadecylimidazole (C_16_-Im).** Imidazole (0.5 g; 7.3 mmol; 1 eq; Sigma-Aldrich) and potassium hydroxide (0.62 g, 11.0 mmol, 1.5 eq) were added to a round-bottom flask and dissolved in DMSO (12 mL; MERCK). The reaction mixture was left at 70°C under magnetic stirring for 30 min, after which either 1-bromotetradecane (2.18 mL; 7.3 mmol; 1.0 eq; Sigma-Aldrich) or 1-bromohexadecane (2.26 mL, 7.3 mmol, 1.0 eq; Sigma-Aldrich) was slowly added. The reaction was allowed to proceed under magnetic stirring at 70°C for 5 h, then the mixture was cooled down to room temperature (r.t.), and water (30 mL) was added. After placing this mixture in an ice bath, a white solid was formed and next filtered, washed with water (3 × 250 mL), and dried under vacuum overnight to afford the final 1-alkylimidazoles in 88% (C_14_-Im) and 84% (C_16_-Im) yield. The structures of these final compounds were confirmed by ^1^H-NMR and ^13^C-NMR, according with the spectral data below. The multiplicity of ^1^H-NMR signals is given as: s, singlet; d, doublet; t, triplet; m, unresolved multiplet; q, quintuplet.

*(a) 1-tetradecyl-imidazole.* Yellow oil (1.71 g, 88%); δ_H_/ppm (400 MHz, CDCl_3_) 7.46 (s, 1H), 7.02 (s, 1H), 6.90 (s, 1H), 3.89 (t, 2H, *J *= 7.6 Hz), 1.73 (q, 2H, *J *= 7.0 Hz), 1.27-1.22 (m, 22H), 0.85 (t, 3H, *J *= 6.9 Hz); δ_C_/ppm (100 MHz, CDCl_3_) 137.1, 129.2, 118.8, 47.2, 32.0, 31.1, 29.7-29.4, 26.6, 22.7, 14.2;

*(b) 1-hexadecylimidazole.* White solid (1.81 g, 84%); δ_H_/ppm (400 MHz, CDCl_3_) 7.57 (s, 1H), 7.06 (s, 1H), 6.90 (s, 1H), 3.93 (t, 2H, *J *= 7.1 Hz), 3.53 (q, 2H, *J *= 7.1 Hz), 1.29-1.24 (m, 26H), 0.87 (t, 3H, *J *= 6.9 Hz); δ_C_/ppm (100 MHz, CDCl_3_) 137.0; 128.8; 119.0; 47.4; 32.0; 31.1; 29.8-29.1; 26.6; 22.8; 14.2;

**(ii) 1-methyl-3-(prop-2-enyl)imidazolium bromide (Pr-MeIm), 1-tetradecyl-3-(prop-2-enyl)imidazolium bromide (Pr-C_14_Im), and 1-hexadecyl-3-(prop-2-enyl)imidazolium bromide (Pr-C_16_Im).** An 80% solution of propargyl bromide in toluene (250 μL; 2.24 mmol, 1.0 eq; Fluorochem) was slowly added to a round-bottom flask containing 1-methyl-imidazole (Alfa Aesar; 195 μL, 2.5 mmol, 1.1 eq), 1-tetradecyl-imidazole (0.664 g, 2.5 mmol, 1.1 eq) or 1-hexadecylimidazole (0.723 g, 2.5 mmol, 1.1 eq), and the mixture kept under magnetic stirring at 40°C for 24 h. After cooling down the yellow oily mixture formed to r.t., dichloromethane (DCM, 4 mL; CARLO ERBA) was added, followed by addition of cold diethyl ether (10 mL; Fisher Chemical). The suspension was placed in an ice bath, occurring precipitation of a white solid that was filtered and washed with diethyl ether (10 mL). This solid was re-dissolved and re-precipitated three times upon alternate addition of 5-mL portions of DCM and 10-mL portions of diethyl ether, to afford the final products in 77% (Pr-MeIm), 45% (Pr-C_14_Im) and 97% (Pr-C_16_Im) yields. The structures of the final alkylimidazolium ILs were confirmed by ^1^H-NMR, ^13^C-NMR, and ESI-IT MS, according with the spectral data below. The multiplicity of ^1^H-NMR signals is given as before.

*(a) 1-tetradecyl-3-(prop-2-enyl)imidazolium bromide.* White solid (0.3887 g, 45.2%); δ_H_/ppm (400 MHz, CDCl_3_) 10.45 (s, 1H), 7.63 (t, 1H, *J *= 1.6 Hz), 7.40 (t, 1H, *J *= 1.6 Hz), 5.44 (d, 2H, *J *= 2.6 Hz), 4.31 (t, 2H, *J *= 7.5 Hz), 2.72 (t, 1H, *J *= 2.6 Hz), 1.91(q, 2H, *J*= 7.4 Hz), 1.33-1.23 (m, 22H), 0.86 (t, 3H, *J *= 6.9 Hz); δ_C_/ppm (100 MHz, CDCl_3_) 137.1; 122.0; 121.9; 77.9; 74.3; 50.6; 40.1; 32.0; 30.3; 29.7-29.4; 26.4; 22.8; 14.2; (EI^+^) *m/z* calculated for C_20_H_35_N_2_^+^: 303.28; found: 303.87 [M]^+^; 687.47 [2M+Br]^+^.

*(b) 1-hexadecyl-3-(prop-2-enyl)imidazolium bromide.* White solid (0.634 g, 97%); δ_H_/ppm (400 MHz, CDCl_3_) 10.5 (s, 1H), 7.65 (s, 1H), 7.43 (s, 1H), 5.45 (d, 2H, *J *= 3.0 Hz), 4.31 (t, 2H, *J *= 7.5 Hz), 2.72 (t, 1H, *J *= 2.4 Hz), 1.90 (q, 2H, J = 7.1 Hz), 1.33-1.23 (m, 26H), 0.85 (t, 3H, J = 6.9 Hz); δ_C_/ppm (100 MHz, CDCl_3_) 137.1, 122.1, 122.0; 77.9, 74.3, 50.6, 40.1, 32.0, 30.3, 29.8-29.4, 29.1, 26.4, 22.8, 14.2; (EI^+^) *m/z* calculated for C_22_H_39_N_2_^+^: 331.31, found 331.50 [M]^+^; 743.24 [2M+Br]^+^.

**(iii) On-resin peptide sequence assembly.** All peptide derivatives based on the amino acid sequence of the parent PP4 (amino acid sequence KTTKS), and this reference peptide itself, were assembled by standard SPPS procedures using the Fmoc/*^t^*Bu orthogonal protection scheme (49). The Fmoc-Rink-amide MBHA resin (100 to 200 mesh, 0.52 mmol/g; NovaBiochem) was used as solid support for the assembly of all peptides except for peptide C_16_-KTTKS-*OH* that was assembled on a preloaded Fmoc-Ser(*^t^*Bu)-Wang resin (100 to 200 mesh, 0.69 mmol/g; NovaBiochem). In all cases, the commercial Fmoc-protected resin was first swelled in *N*,*N*-dimethylformamide (DMF; CARLO ERBA) for 30 min and next treated with piperidine (20%; MERCK) in DMF for 20 min at r.t., for removal of the Fmoc protecting group. After washing steps using DMF and DCM (3 × 10 mL), coupling of the desired amino acid residue was performed using a mixture of the Fmoc-protected amino acid (5 eq; Bachem), *O*-(benzotriazol-1-yl)-*N*,*N*,*N′*,*N′*-tetramethyluronium hexafluorophosphate (HBTU, 5 eq; NovaBiochem), and *N*-ethyl-*N*,*N*-diisopropylamine (DIEA, 10 eq; VWR) in DMF, which was added to the resin and kept at r.t for 1 h, under stirring. The deprotection and coupling cycles were repeated under the same conditions until the full amino acid sequence was assembled and, whenever desired, the Fmoc-Lys(Boc)-OH (NovaBiochem) standard building block was replaced by its ε-azide analogue, Fmoc-Lys(N_3_)-OH. For the synthesis of the *N*-terminally modified peptides, namely, 2-azidoethanoyl-KTTKS (N_3_-KTTKS) and the reference palmitoyl peptides C_16_-KTTKS-*NH_2_* and C_16_-KTTKS-*OH*, the same general procedure was adopted, with an additional sequence elongation step using, respectively, 2-azidoacetic (37.5 μL, 0.5 mmol, 5 eq; Sigma-Aldrich) or palmitic (0.128 mg, 0.5 mmol, 5 eq; Sigma-Aldrich) acids instead of a Fmoc-protected amino acid.

**(iv) On-resin “click” reaction (CuAAC).** While still anchored to the resin, azide-modified peptides were further reacted with the relevant propargyl-ILs *via* CuAAC. To this end, a solution containing the relevant propargyl-IL (Pr-MeIm) (20 mg, 0.1 mmol, 1 eq), Pr-C_16_Im (53 mg, 0.12 mmol, 1.2 eq) or Pr-C_14_Im (38.3 mg, 0.1 mmol, 1eq), 2,6 lutidine (116 μL, 1 mmol, 10 eq; Alfa Aesar), sodium L-ascorbate (19.8 mg, 0.1 mmol, 1 eq; Sigma-Aldrich), and DIEA (170 μL, 1 mmol, 10 eq; VWR) in DMF (3 mL) was added to the desired azido-peptidyl resin, followed by addition of copper(I) bromide (14.3 mg, 0.1 mmol, 1 eq; Fluka) in ACN (1 mL; CARLO ERBA). The “click” reaction was allowed to proceed at r.t. under stirring for 24 h. After draining the resin, this was thoroughly washed with 0.1 M aqueous ethylenediaminetetraacetic acid (EDTA, 5 × 10 mL; PanReac AppliChem) to ensure full removal of copper, followed by DMF (3 × 10 mL) and DCM (3 × 10 mL), to wash out all other unreacted materials and side products.

**(v) Cleavage and purification of final peptides and their IL conjugates.** Once fully assembled, the peptides were fully deprotected and released from the solid support through a 2h acidolysis using a cleavage cocktail containing 95% of TFA (VWR), 2.5% of triisopropylsilane (TIS; Alfa Aesar), and 2.5% of deionized water. The crude peptide material thus obtained was next purified by RP-HPLC on a Hitachi-Merck LaPrep Sigma system equipped with an LP3104 UV detector and an LP1200 pump, using an RP-C_18_ column (250 × 25 mm, 5 μm pore size) and an elution gradient using 0.05% aqueous TFA as solvent A and acetonitrile (ACN) as solvent B. The pure peptide fractions were collected, pooled, and freeze-dried to afford the final peptide as a fluffy white solid.

**(vi) Quantitation of final peptides and their IL conjugates.** Peptide stock solutions were prepared at approximately 10 mg/mL in distilled water except for C_16_-KTTKS-*OH* that was solubilized in DMSO. Accurate quantitation of the peptide solutions were quantitated by microvolume spectrophotometry at 205 nm, using a Thermo Scientific NanoDrop One system and quantitation method 31 that assumes an extinction coefficient ε_205_ of 31 mL·mg^−1^·cm^−1^ (50). Exception was made for the reference peptide C_16_-KTTKS-*OH*, which was quantified through amino acid analysis (AAA) due to its poor solubility in water, making it inadequate for NanoDrop. To this end, the peptide was hydrolyzed using 6 M aqueous hydrochloric acid containing phenol (10% vol/vol), for 24 h at 110°C. The hydrolysate was next evaporated to dryness and the residue dissolved in HPLC-grade water (500 μL) containing α-aminobutyric acid as internal standard. The mixture thus obtained was then derivatized by the AccQ-Tag protocol from Waters using 6-aminoquinoyl-*N*-hydroxysuccinimidyl carbamate (51), and the resulting solution analyzed by HPLC (WATERS 600) with the UV-detector (WATERS 2487) set at 254 nm.

### *In vitro* assays.

**(i) Antibacterial activity in standard conditions.** The test conjugates and reference compounds were evaluated for their activity against three reference strains of Gram-negative bacteria, namely, E. coli (ATCC 25922), Pseudomonas aeruginosa (ATCC 27853), and Klebsiella pneumoniae (ATCC 13883), and the four following reference strains of Gram-positive bacteria, Staphylococcus aureus (ATCC 29213), S. Epidermidis (ATCC 14990), E. faecalis (ATCC 29212), and Streptococcus pyogenes (ATCC 19615). MIC values were assessed using the broth microdilution method in cation adjusted MHB2 - Sigma-Aldrich, except for S. pyogenes (a group A beta-hemolytic Streptococcus), where the medium was previously supplemented with lysed horse blood (Sigma-Aldrich) at 2.5% according to the CLSI guidelines (28). MBC values were also determined as previously reported (52). The two conjugates displaying higher antibacterial activity were further evaluated by determining their MIC against MDR clinical isolates of K. pneumoniae (KP010), P. aeruginosa (PA004), and S. aureus (SA007).

**(ii) Antibacterial activity in simulated wound fluid.** MIC values of the most active conjugates, C_16_Im-KTTKS and KTTK(C_16_Im)S, were also determined in SWF against S. aureus (ATCC 29213). Briefly, the conjugates were dissolved in water and diluted in 0.02% aqueous acetic acid containing 0.4% of bovine serum albumin (BSA; Sigma-Aldrich) to final concentrations ranging from 1,280 to 1.25 μg/mL. In parallel, SWF was prepared to a final composition of 50% fetal bovine serum (FBS; Sigma-Aldrich) and 50% peptone water (0.9% NaCl in 0.1% aqueous peptone; Sigma-Aldrich) (35). S. aureus was incubated (10^5^ CFU/mL) in either MHB or SWF in the presence of the test conjugates at concentrations between 128 and 0.125 μg/mL, and bacterial growth assessed after 18 h of incubation at 37°C. MIC values were determined in triplicates and are a result of three independent experiments. MBC values were also determined by plating 10 μL of the content of the first three wells where bacterial growth was not observed, followed by incubation in Tryptic Soy Agar (TSA; Sigma-Aldrich) for 24 h at 37°C.

**(iii) Antifungal activity.** The two conjugates with stronger antibacterial action, and relevant parent compounds and their mixture, were also tested for antifungal activity, using three reference strains of *Candida* spp., namely, C. albicans ATCC 90028, C. glabrata ATCC 90030, and C. parapsilosis ATCC 22019. MIC values were determined using a broth microdilution method in RPMI 1640, supplemented with glucose to a final concentration of 2% (RPMI 2% G), according to the EUCAST protocol (36).

**(iv) Toxicity to human cells.** HaCat and HFF-1 were seeded at 4 × 10^4^cell/mL in 96-well plate using in Dulbecco’s Modified Eagle Medium (DMEM; CLS) supplemented with 2% FBS (Biowest) and 1% of antibiotic/antimycotic solution (100 units/mL of penicillin, 10 mg/mL of streptomycin, and 0.25 mg/mL of amphotericin B, Sigma-Aldrich). The plate was incubated at 37°C in a 5% CO_2_ atmosphere, and the cells allowed to grow until confluence was reached. Then, solutions of the test compounds in DMEM supplemented with 2% FBS, in a concentration range between 6.3 and 100 μM, were added to the wells. After 24 h of incubation at 37°C in a 5% CO_2_ atmosphere, the cell viability was assessed through the resazurin reduction assay. Briefly, the medium was removed and then 20 μL of the alamarBlue Cell Viability Reagent (Resazurin sodium salt, Sigma-Aldrich) at 0.15 mg/mL in 100 μL of Hank’s Balanced Salt Solution (HBSS; Sigma-Aldrich) were added to each well; the plate was next incubated for 2 h at 37°C in a 5% CO_2_ atmosphere, after which fluorescence was read at 560/590 nm on a Flex Station 3 multi-mode microplate reader (Molecular Devices) (53). The IC_50_ values were thus determined using the GraphPad Prism 9.0 software, by applying the equation log(inhibitor) *versus* response with variable slope (four parameters).

**(v) Collagen production in human dermal fibroblasts.** The amount of collagen produced by HDF was determined using the SIRCOL Kit Assay (Biocolor) according to the manufacturers’ instructions (38). Briefly, the cells were seeded in 6-well plates, using DMEM supplemented with 10% FBS and 1% antibiotic/antimycotic solution. When confluence was achieved, DMEM supplemented with 2% of FBS and containing the test conjugates at 5 μM was added to the cell cultures, which were then incubated over 48 h at 37°C in a 5% CO_2_ atmosphere. Medium was then removed, and quantitation of the newly formed collagen proceeded as follows: collagen was solubilized in cold 0.5 M aqueous acetic acid (Fisher Chemical) and concentrated overnight with deposition of a transparent pellet; after centrifugation, the supernatant was discarded and the pellet was labeled with the SIRCOL dye reagent; after removing the unbound dye with the acid-salt wash reagent, the collagen-bound dye was dissolved with the alkali reagent and absorbance was measured at 555 nm on Flex Station 3 multimode microplate reader. The collagen concentration was then determined by interpolation in a standard curve built by using the same quantitation method on standard solutions of rat collagen in 0.5 aqueous acetic acid.

Results were expressed as mean ± standard error of mean (SEM) values for collagen amount (% of control) and the statistical analysis was performed in GraphPad Prism 9.0.0 Software using T student, paired test, *P* values two-tailed with confidence interval of 95% (*, *P* < 0.05; **, *P* < 0.01).
