# Erratum for Gomes et al., “Boosting Cosmeceutical Peptides: Coupling Imidazolium-Based Ionic Liquids to Pentapeptide-4 Originates New Leads with Antimicrobial and Collagenesis-Inducing Activities”

**DOI:** 10.1128/spectrum.00443-24

**Published:** 2024-06-24

**Authors:** Ana Gomes, Lucinda J. Bessa, Iva Fernandes, Luísa Aguiar, Ricardo Ferraz, Cláudia Monteiro, M. Cristina L. Martins, Nuno Mateus, Paula Gameiro, Cátia Teixeira, Paula Gomes

## ERRATUM

Volume 10, no. 4, e02291-21, 2022, https://doi.org/10.1128/spectrum.02291-21. Page 12, Acknowledgments: The statement “We declare no conflicts of interest” should be removed.

